# New infundibulopelvic angle measurement method can predict stone-free rates following retrograde intrarenal surgery

**DOI:** 10.1038/s41598-024-60248-7

**Published:** 2024-04-30

**Authors:** Yu-Hung Tung, Wei‑Ming Li, Yung-Shun Juan, Tsung-Yi Huang, Yen-Chun Wang, Hsin-Chih Yeh, Hsiang-Ying Lee

**Affiliations:** 1 Department of Urology, Kaohsiung Medical University Hospital, No. 100, Shih-Chuan 1st Road, Sanmin Dist., Kao-hsiung, 80708 Taiwan; 2 Department of Urology, School of Medicine, College of Medicine, Kaohsiung Medical University, Kao-hsiung, Taiwan; 3 Department of Urology, Ministry of Health and Welfare Pingtung Hospital, Pingtung, Taiwan; 4 Department of Urology, Kaohsiung Medical University Gang-Shan Hospital, Kao-hsiung, Taiwan; 5 Graduate Institute of Clinical Medicine, College of Medicine, Kaohsiung Medical University, Kao-hsiung, Taiwan; 6 Department of Urology, Kaohsiung Municipal Ta-Tung Hospital, Kao-hsiung, Taiwan

**Keywords:** Renal calculi, Anatomy

## Abstract

To enhance the accuracy of predicting stone-free rates after retrograde intrarenal surgery, we devised a novel approach to assess the renal infundibulopelvic angle. We conducted a retrospective review of patient records for those who underwent retrograde intrarenal surgery for renal stones between April 2018 and August 2019. Patient demographics, stone characteristics, and perioperative data were recorded. Subsequently, we introduced a modified angle measurement called the pelvic stone angle and evaluated its predictive performance for stone-free rates by comparing it with the traditional method in scoring systems. A total of 43 individuals were included in this study. Notable differences in stone burden and Hounsfield unit measurements were found between stone-free and non-stone-free patients. The pelvic stone angle demonstrated a good model fit when used in scoring systems, performing equally well as the conventional approach. The area under the receiver operating characteristic curve for the R.I.R.S. scoring system using the pelvic stone angle and the conventional approach did not show a significant difference. In conclusion, the predictive ability of the pelvic stone angle for stone-free rates was comparable to the old measurement method. Moreover, scoring systems using the pelvic stone angle exhibited a better model fit than those using the conventional approach.

## Introduction

Nephrolithiasis is a highly prevalent disease with an increasing incidence worldwide in recent years^1^. Extracorporeal shock wave lithotripsy (SWL), ureteroscopic lithotripsy, and percutaneous nephrolithotomy (PCNL) are common interventional therapies according to the American Urological Association (AUA) guidelines^2^. Retrograde intrarenal surgery (RIRS) has become increasingly popular in recent years owing to technological advancements, including the introduction of single-use flexible ureteroscopes, improved visual systems, enhanced deflection mechanisms, and sophisticated lithotripsy probes. RIRS has shown promising results in treating renal stones, even in cases where previous studies have indicated lower stone-free rates (SFR). This includes cases involving stones larger than 2 cm, those situated in the lower pole, and multiple multicalyceal stones^3–5^. SFR after treatment with RIRS is associated with multiple factors such as stone burden, stone density, and localization^6–8^. Moreover, retrograde ureteroscopic access to renal stones in the lower pole is challenging and various unfavorable anatomical features should be considered, including small renal infundibulopelvic angle (RIPA) and long infundibular length (IL)^9,10^. Several scoring systems based on these factors have been developed and externally validated recently. However, adoption of these scoring systems in clinical practice is limited. Possible reasons for this could be the lack of widespread validation, consistency, practicality and feasibility issues^11–14^. Additionally, different measurement methods of RIPA and the original definition, which measures the angle on intravenous pyelogram (IVP) rather than currently and frequently used non-contrast-enhanced computed tomography (NCCT), also lead to confusion for physicians. Moreover, with benefits of decreasing intrarenal pressure, improving visibility, and providing easy access to the renal pelvicocalyceal system, the widely used ureteral access sheath (UAS) during RIRS also has an impact on the measurement of RIPA. In this study, we aimed to develop an innovative measuring method of renal infundibulopelvic angle more suited to the current RIRS procedure. We then compared the capabilities of this new method with the existing one.

## Methods

### Data collection

We retrospectively reviewed the database records of patients with renal stones who underwent RIRS procedure April 6, 2018, and August 20, 2019. To evaluate the new measuring method of RIPA, we specifically selected patients who had at least one kidney stone located in the lower pole, and these patients may also have stones in other calices of the kidney. Our study did not have specific stone size inclusion criteria. The study excluded specific cases with rare conditions during the study period, as their limited numbers could potentially distort the true nature of the research. Patients were excluded according to the following exclusion criteria: (1) patients aged < 18 years; (2) patients undergoing procedures that were concomitantly performed with other surgeries; (3) patients with special situations such as pregnancy, duplicate ureteral deformity and horseshoe kidney; and (4) patients who were pre-stented.

### Definition of new measuring method: pelvic stone angle

**Figure 1:**
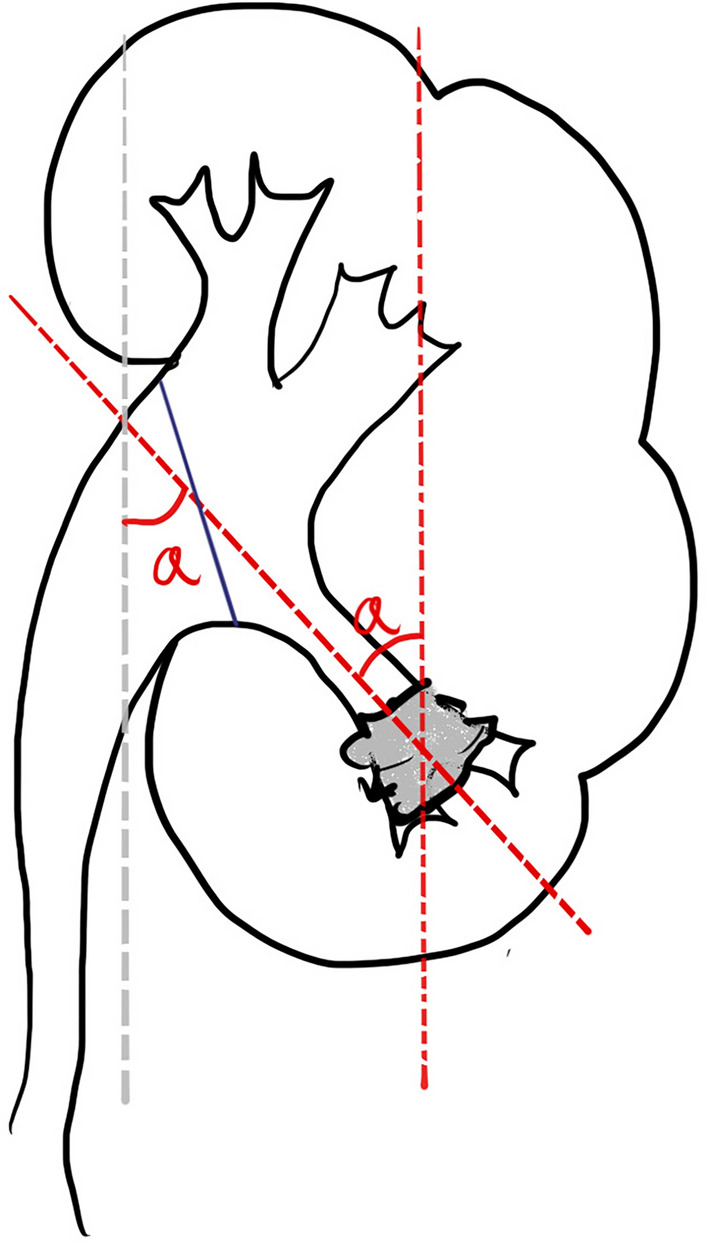
Illustration of pelvic stone angle (indicated by angle ‘a’). The gray dashed line (representing the ureteral axis with the ureteral sheath in place) is parallel to the vertical line passing through the stone, ensuring that the two angles, denoted as “a”, are equal. This alignment further signifies the angle that RIRS procedures need to overcome.

The new measuring method involves drawing a vertical line passing through the center of the lower pole stone and then drawing a line connecting the center point of the stone to the midpoint between the highest and lowest points of the ureteropelvic junction. The angle formed by these two lines is referred to as the pelvic stone angle (PSA), as shown in Fig. 1, indicated by angle ‘a’. The infundibulopelvic angle (IPA) represents the previously used method for measuring the RIPA, as proposed by Elbahnasy^15^.

### Evaluation factors

We collected general information (age, sex, body mass index (BMI), comorbidities, pre-operative hemoglobin, estimated glomerular filtration rate, and creatinine) as well as characteristics of the renal stones (location, size, stone number, and mean computed tomography (CT) attenuation number). Additionally, we specifically recorded the characteristics of the lower pole stones, including CT attenuation number and stone burden.

All patients underwent NCCT scans before the surgery to obtain anatomic features, including PSA, IPA, IL and infundibular width (IW). Stone burden was calculated as cumulative stone diameter (CSD). The average Hounsfield Unit (HU) value of the stones was determined by calculating the mean CT attenuation values at both the center and outermost edges of each stone. In patients with multiple stones, stone diameter was calculated by summing the diameter of each individual stone. In contrast, the mean HU value was determined by averaging the mean HU value of each stone present in the patient. Multifocal is defined as a condition in which the stone's location involves more than one calyx. Stone-free status was defined as the absence of any evidence of stones or stone fragments less than 1 mm on kidney-ureter-bladder (KUB) X-ray or CT scan taken one month after the RIRS. All imaging-related data mentioned above were obtained by the independent evaluation of images by two physicians. The data sets generated and analyzed during the current study are available from the corresponding author on reasonable request.

### Retrograde intrarenal surgery technique

All surgeries were conducted by experienced surgeons using various sizes and brands of ureterorenoscopes, including Richard Wolf and Boston Scientific, with sizes ranging from 6 to 8 Fr. For UAS, we employed products from COOK Medical and Boston Scientific, available in sizes 11/13Fr. and 12/14Fr. The procedures were performed under general anesthesia, with the patients in the lithotomy position. Prior to stone fragmentation, diagnostic ureteroscopy was performed using a semi-rigid ureterorenoscope to examine the ureter and insert a safety guidewire. Subsequently, a UAS was placed to facilitate stone extraction and reduce intrarenal pressure^16^. A flexible ureteroscope was then inserted through the UAS and the stones were fragmented using a holmium:YAG laser. In some cases, tipless nitinol stone baskets were used to remove certain stone fragments. At the conclusion of the procedure, the decision to place a D-J stent was made at the surgeon's discretion.

### Assessing the utility of pelvic stone angle (PSA)

To evaluate the clinical utility of PSA in predicting the SFR after RIRS, we compared it with existing scoring systems, including the R.I.R.S. scoring system and the Resorlu-Unsal stone score (RUSS). Both of these systems incorporate RIPA as a significant influencing factor and have demonstrated good predictive ability for SFR after RIRS^17,18^. Several studies have externally validated the clinical effectiveness of these scoring systems^13,19,20^.

Therefore, we replaced IPA with PSA in the scoring systems, hoping that PSA would exhibit a predictive performance equivalent to or even surpassing IPA. The objective was to determine if PSA can serve as an effective and comparable alternative to IPA in predicting SFR following RIRS.

### Statistical analysis

T-test was used to assess the correlation between dependent variables and continuous variables with normal distribution, and the Mann–Whitney U test was for non-normal continuous variables. Correlations between dependent variables and categorical variables were assessed using Pearson Chi-square test or Fisher’s exact test. The potential correlation between scoring systems and stone free was analyzed by univariate and multivariate logistic regression. We evaluated the model fitting for different scoring systems with Area under the curve (AUC), Akaike information criterion (AIC) and Bayesian information criterion (BIC), and the nonparametric approach developed for generalized U-statistics was used to compare different receiver operating characteristic (ROC) curves. Accuracy indicators presented the classification performance of the scoring system models. We conducted a logistic regression model to evaluate scoring system correctly predicted probability of stone free, and performed the multiple comparisons. The *P* value was adjusted by Tukey–Kramer and Bonferroni method in post-hoc test. Regression coefficients showed the correlation of independent variables with perioperative data and recurrence. Statistical significance was set at *P* < 0.05. Statistical analysis was performed using SAS (version 9.4, North Carolina State University, USA).

### Ethical statement

The study was conducted by the Helsinki Declaration (as revised in 2013). All procedures performed in this study were approved by the Ethics Committee of the Kaohsiung Medical University (KMUHIRB-F(I)-20,190,129). The requirement for informed consent was waived by the Ethics Committee of the Kaohsiung Medical University (KMUHIRB-F(I)-20,190,129).

## Results

A total of 43 individuals who underwent RIRS and laser lithotripsy between April 2018 and August 2019 were included in the study. Table 1 present a comparison of the characteristics and demographics between stone-free and non-stone-free patients. Significant differences were observed between the two groups in terms of lower pole stone burden, overall renal stone burden, overall mean CT attenuation number, scores of the R.I.R.S. scoring system (using both IPA and PSA), and the presence of multifocal stones.

**Table 1:** Demographics of patients and characteristics of stones and scoring systems according to stone-free status.

	Non-SF	SF	*P* value
Patients, n	22	21	
Sex, n (%)			0.253
Male	15 (68)	15 (71)	
Female	7 (32)	6 (29)	
Age, years, median (SD)	57.27 (± 11.36)	55.9 (± 10.42)	0.6833
Hypertension, n (%)	11 (50)	13 (62)	0.1793
Diabetes mellitus, n (%)	6 (27)	11 (52)	0.0625
BMI, kg/m^2^, median (SD)	27.44 (± 4.66)	26.05 (± 3.89)	0.2943
Hydronephrosis, n (%)	10 (45)	9 (43)	0.2374
Hospitalization, days, median (IQR)	4 (3–7)	3 (3–3)	0.0015
Smoking, n (%)	4 (18)	1 (5)	0.1596
Lower pole stone			
Stone burden, cm (IQR)	1.7 (1.1–2.27)	1.06 (0.75–1.5)	0.0136
CT number, HU (SD)	1036.39 (± 352.12)	825.95 (± 369.26)	0.0628
Overall stone burden, cm (IQR)	2.1 (1.6–3.1)	1.3 (0.9–1.8)	0.0026
Overall CT number, HU (SD)	1066.73 (± 298.52)	829.52 (± 361.83)	0.0237
Anatomical features			
PSA, degree (SD)	50.93 (± 12.48)	47.98 (± 8.32)	0.3685
IPA, degree (SD)	45.4 (± 20.18)	42.23 (± 19.64)	0.6051
RIL, mm (SD)	25.32 (± 5.72)	24.49 (± 4.88)	0.6114
IW, mm (IQR)	4.6 (4.1–5.4)	4.1 (2.9–6.3)	0.3492
Multifocal of stones, n (%)	10 (45)	6 (28)	0.0425
Numbers of stone			0.0962
Multiple, n (%)	13 (59)	8 (38)	
Recurrence, n (%)	6 (27)	3 (14)	0.176
RUSS score			
IPA, points (IQR)	2 (1–2)	1 (1–1)	0.0861
PSA, points (IQR)	1.5 (1–2)	1 (0–1)	0.0583
R.I.R.S. score			
IPA, points (IQR)	7 (6–8)	6 (5–6)	0.0042
PSA, points (IQR)	7 (6–7)	6 (5–6)	0.0032

*SF* Stone-free, *BMI* Body mass index, *CT* Computed tomography, *HU* Hounsfield unit, *PSA* Pelvic stone angle, *IPA* Infundibulopelvic angle, *RIL* Renal infundibular length, *IW* Infundibular width, *RUSS* Resorlu-Unsal stone score, *SD* Standard deviation, *IQR* Interquartile range.

**Figure 2:**
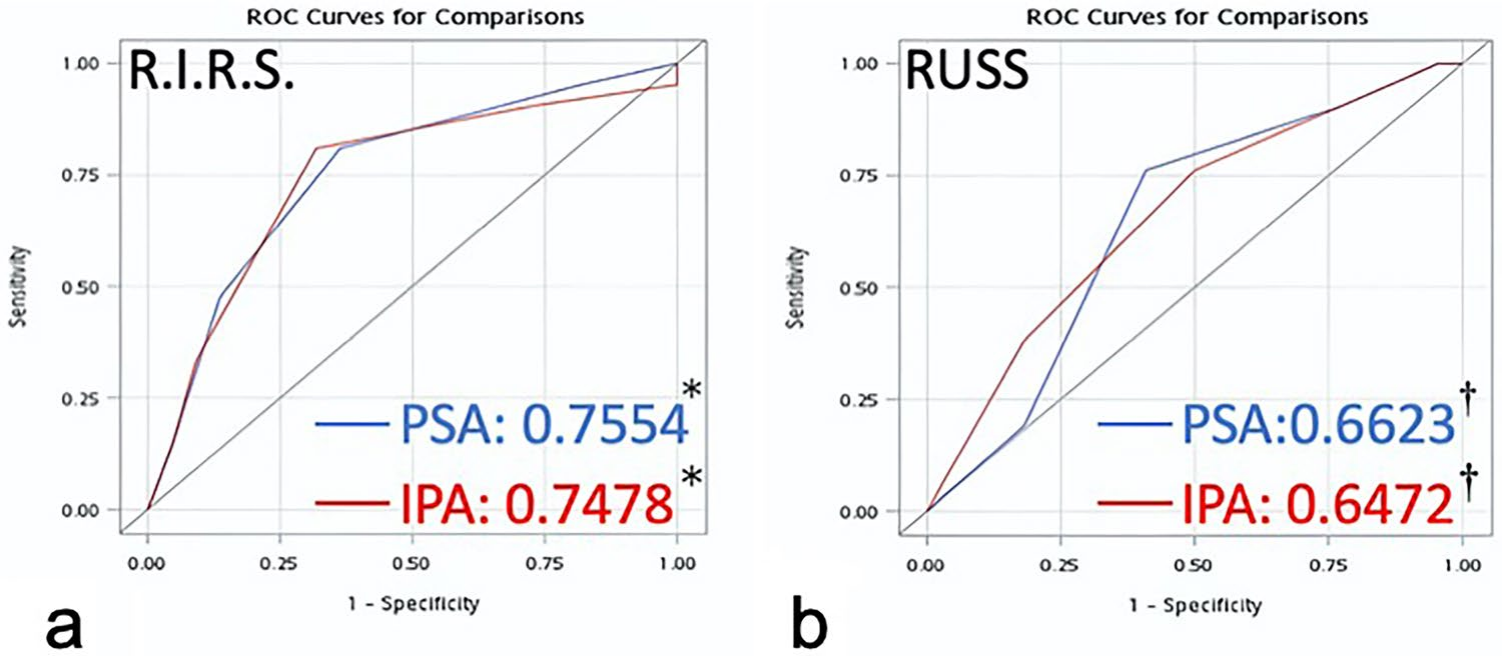
The predictive capability of scoring systems using different angle measurement methods for stone-free rates. (**a**) The ROC curves and *AUC of R.I.R.S. scoring system using PSA (blue line) and IPA (red line). (**b**) The ROC curves and †AUC of RUSS using PSA (blue line) and IPA (red line).

In the univariate logistic regression model, stone burden and HU were found to be associated with the SFR. The relationships were both statistically significant when considering stone-free status and R.I.R.S. score using IPA [OR (95% CI): 0.471 (0.254–0.871)] and PSA [OR (95% CI): 0.406 (0.209–0.788)] as parameters. Irrespective of whether R.I.R.S. score or RUSS score was considered, the PSA demonstrated a good model fitting that was not inferior to using IPA (Table 2). In the R.I.R.S. scoring system, the AUC for predicting SFR using PSA is 0.7554, while with IPA, it is 0.7478. Additionally, there was no difference in the AUC comparison test using Delong's method (Table 2 and Fig. 2). The accuracy of the R.I.R.S. scoring systems when using PSA and IPA for predicting stone-free outcomes was both greater than 0.7, with accuracy indicators presented in Table 3.

**Table 2:** Model selection and AUC comparison between R.I.R.S. scoring system and RUSS with PSA and IPA inputs.

	IPA	PSA
RUSS		
AIC	60.47	59.614
BIC	63.992	63.137
AUC (95% CI)	0.6472 (0.4829–0.8115)	0.6623 (0.5049–0.8197)
AUC difference (95% CI)	0.0152 (− 0.0872–0.1175)	
*P value	0.7717	
R.I.R.S		
AIC	56.391	54.543
BIC	59.914	58.065
AUC (95% CI)	0.7478 (0.5977–0.8980)	0.7554 (0.6110–0.8998)
AUC difference (95% CI)	0.00758 (− 0.0438–0.0590)	
*P value	0.7726	

*PSA* Pelvic stone angle, *IPA* Infundibulopelvic angle, *RUSS* Resorlu-Unsal stone score, *AIC* Akaike information criterion, *BIC* Bayesian information criterion, *AUC* Area under the curve.

*This *P* value indicates whether there is a statistically significant difference in the AUC between the R.I.R.S. scores and RUSS scores calculated using IPA and PSA, respectively.

**Table 3:** Performance metrics comparison for scoring systems using IPA and PSA to predict stone-free rate.

	Sensitivity	Specificity	PPV	NPV	Accuracy
*RUSS					
IPA	0.6400	0.7222	0.7619	0.5909	0.6744
PSA	0.5926	0.6875	0.7619	0.5000	0.6279
*R.I.R.S					
IPA	0.7083	0.7895	0.8095	0.6818	0.7442
PSA	0.6800	0.7778	0.8095	0.6364	0.7209

*PSA* Pelvic stone angle, *IPA* Infundibulopelvic angle, *PPV* Positive predictive value, *NPV* Negative predictive value, *RUSS* Resorlu-Unsal stone score.

*R.I.R.S. score and RUSS score calculated using IPA and PSA, respectively.

## Discussion

According to the AUA guidelines on urolithiasis^2^, for kidney stones larger than 20 mm, PCNL remains the first-line treatment. Although PCNL is more effective, it is associated with a higher complication rate and a longer hospital stay^21^. In cases where PCNL is contraindicated or not preferred by the patient, RIRS can be considered an alternative option^4^. Additionally, with significant technological advancements in the field of RIRS, studies have indicated that RIRS can also achieve successful treatment of kidney stones even those larger than 30 mm^21,22^.

Lower pole stones pose a difficulty, and the SFR with SWL is significantly lower^23^. The AUA recommends using PCNL for lower pole stones larger than 1 cm. Some meta-analyses have shown that PCNL has a higher SFR for lower pole stones, but it is often accompanied by a longer hospital stay and higher complication rate. However, RIRS is relatively safer and its SFR is acceptable^23,24^. Therefore, there is no clear superiority between the two methods, and the choice should consider individual factors such as stone characteristics, overall health status, and patient preference.

Moreover, RIRS has some drawbacks, such as higher costs compared to other treatments, primarily due to the fragility of the ureteroscope. This fragility results in increased expenses for items, such as extraction baskets, laser fibers, and access sheaths. Based on the aforementioned reasons, the optimal treatment approach should strike a balance between advantages, potential complications, and overall costs.

Various nephrolithometric scoring systems have been developed to guide treatment decisions and optimize kidney stone management. Several of these systems are currently used to predict SFR in kidney stone management. These include R.I.R.S., RUSS, Ito's nomogram, T.O.HO., and modified S-ReSC^17,18,25–27^. In recent years, numerous studies have conducted external validation and comparison of these scoring systems to determine which one exhibits the best predictive ability for SFR. However, the results in each study, particularly the AUC values, have varied, and no scoring system has been universally considered ideal and most suitable^15,16^.

One reason for this lack of consensus is the variability in the design of each scoring system. The variables included in the scoring system calculations differed, and treatment approaches also varied among different institutions. Additionally, the definition of treatment success (stone-free status), post-operative follow-up period, and imaging modalities differed among the various scoring systems.

In both the R.I.R.S. and RUSS scoring systems, the RIPA is considered a significant factor influencing the SFR, and this viewpoint is also supported by other studies. However, there are different methods for measuring the RIPA, with the most commonly used being the measurement method proposed by Elbahnasy and Sampaio^15,28^. The lack of a unified definition may cause confusion among measurers and further impact the predictive accuracy of the scoring systems.

Moreover, the previous measurement methods were based on intravenous pyelograms rather than the current preoperative imaging modality, NCCT, leading to differences in measurements. Additionally, during RIRS procedures, a UAS is often utilized, which can straighten the ureteral axis and introduce discrepancies between the measured IPA from preoperative imaging and the actual situation during surgery, potentially reducing the accuracy of preoperative assessment.

Therefore, we aimed to establish a new measurement method using NCCT that takes into consideration the use of UAS. This method is referred to as the "pelvic stone angle, as described in the Methods section. This angle represents the degree of bending required by the flexible ureteroscope during the procedure. We have employed a vertical line instead of the previously utilized ureteropelvic axis, as described by Elbahnasy^15^. This decision was prompted by the potential alteration of the ureter’s natural anatomical configuration when a UAS was employed, leading to its almost straightened appearance. Therefore, we expect that this new angle more accurately represents the angle that the flexible ureteroscope needs to overcome during the surgery when using a UAS. Furthermore, as mentioned above, current preoperative assessments rely heavily on NCCT imaging. Therefore, we believe that using the PSA measured from NCCT is more representative and relevant for the assessment and planning of RIRS procedures. To validate the clinical validity and applicability of PSA, we simultaneously calculated the scores of R.I.R.S. and RUSS using both IPA (measured using Elbahnasy’s method) and PSA. We then compared the predictive differences in SFR between the two measurement methods to assess the performance of PSA in comparison to the traditional IPA method.

In this study, the calculated PSA cut-off value influencing stone-free rate was approximately 50 degrees, determined using Youden’s index and ROC curve analysis. However, due to our small sample size, the statistical significance of this finding is debatable. Similarly, IPA cut-off values vary across different studies, with R.I.R.S scoring system and RUSS adopting cut-off points of 30 degrees and 45 degrees, respectively. Additionally, in our study, there was no significant difference between the IPA and PSA measurements within the same patient (*P* value for *t* test = 0.104). Therefore, we directly replaced the IPA with PSA in both scoring systems, maintaining the originally designed cut-off point.

Consistent with previous research findings, the R.I.R.S. scoring system exhibited a better predictive ability for SFR than RUSS^12^. In our study population, the AUC values for the R.I.R.S. scoring system were 0.7478 and 0.7554 when IPA and PSA were used as components (Fig. 2), respectively, with no significant differences observed between the two methods. These results align with the external validation literature^20^, where the AUC for the R.I.R.S. scoring system was reported as 0.737, indicating the representativeness of our study population.

Furthermore, as all patients included in our study had at least one lower pole stone, it was expected that the SFR in our study population would be lower than that in the general population. However, in a study that established the R.I.R.S. scoring system, there were patients with lower pole stones (N = 145) with a SFR of 47.6%^18^, which is similar to our study's SFR of 48.9%. This similarity further reinforces the representativeness of our study population.

Regarding the prediction of SFR in the R.I.R.S. scoring system, IPA and PSA demonstrated accuracies of 0.7442 and 0.7209, respectively, with PSA slightly lower than IPA. However, there was no statistically significant difference between the two (*P* value = 0.8076).

Our findings suggest that IPA and PSA are comparable in predicting SFR, indicating that PSA is equally capable of representing the anatomical characteristics that urologists need to consider when dealing with lower pole stones.

This was a retrospective study, and as such, it was subject to limitations commonly associated with retrospective studies. Additionally, the use of different imaging modalities (NCCT or KUB) for post-operative evaluation of SFR may introduce potential errors and bias, leading to distorted research findings. Second, the study had a relatively small sample size and was conducted at a single center, which may limit the generalizability of the results to a broader population. Consequently, due to these factors, we lack the confidence to determine the PSA cut-off value that influences the stone-free rate. Finally, the R.I.R.S. scoring system was designed to exclude patients with renal anatomical or musculoskeletal abnormalities, which may occur infrequently but are still relevant in clinical practice. In conclusion, the role of this study is that of a preliminary report, aiming to define angles that better align with current clinical practices (such as the widespread use of NCCT and UAS) and incorporate them into scoring systems for predicting post-operative SFR in patients with RIRS. Our results indicate that PSA’s predictive ability for SFR is comparable to IPA. Moreover, scoring systems using PSA as parameter information exhibited better model fit than those using IPA. In the future, further large-scale, multicenter, and multi-cohort external validation will be necessary to confirm the superiority of this angle over traditional IPA measurements.

## Data Availability

The datasets generated during and/or analysed during the current study are available from the corresponding author on reasonable request.
